# Supplementary material for: The interactome of intact mitochondria by cross-linking mass spectrometry provides evidence for coexisting respiratory supercomplexes
Source: Mol Cell Proteomics. 2017 Dec 8;17(2):216–32. doi: 10.1074/mcp.RA117.000470 (PMC5795388; doi:10.1074/mcp.RA117.000470)
Supplement: Supplemental Data [file supp_RA117.000470_133922_0_supp_23978_fzffwf.zip › spectra_annotation/mito_spectra_annotation/282-1-2-1-9-1.pdf]

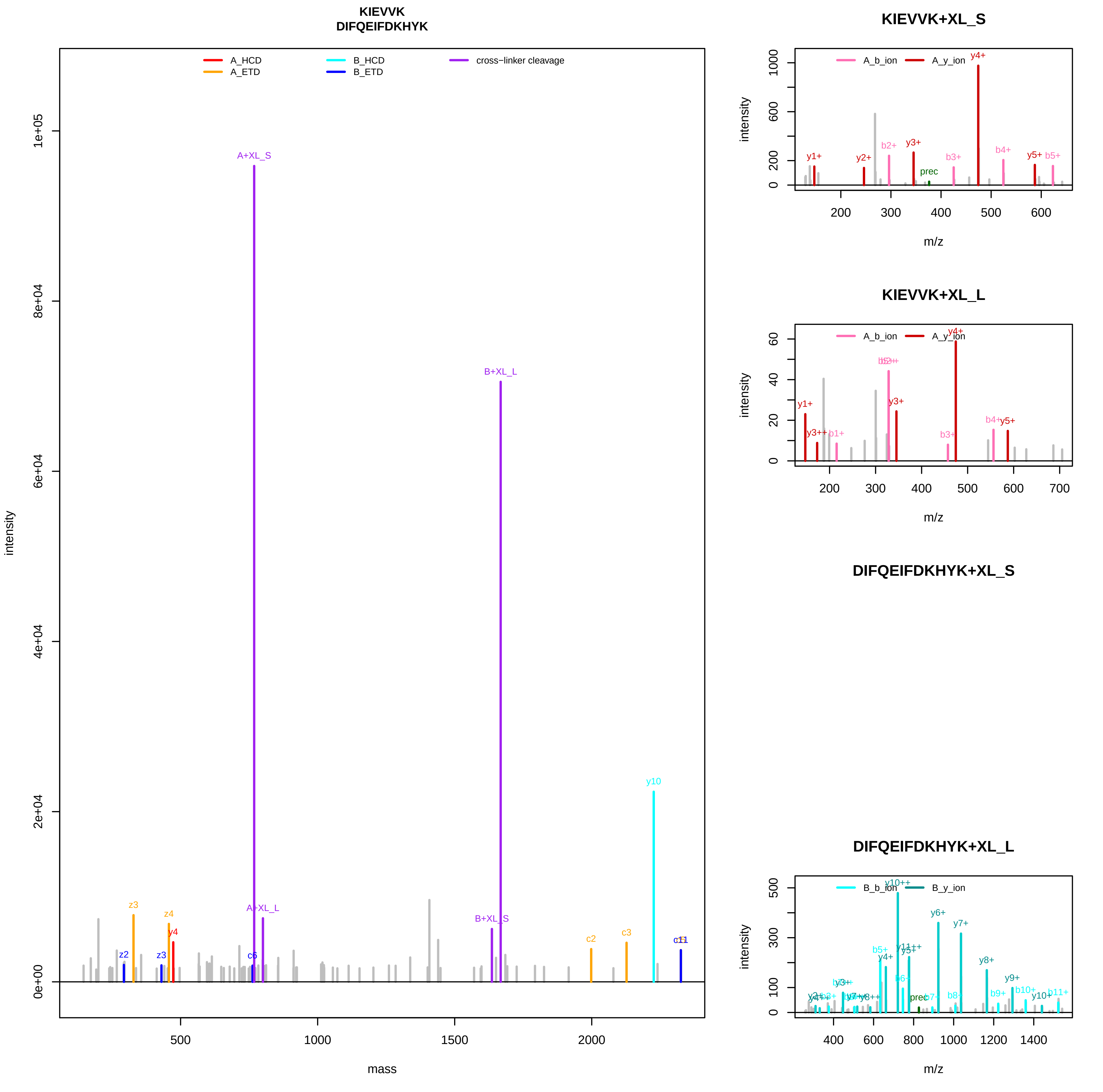

KIEVVK
DIFQEIFDKHYK
A_HCD
A_ETD
B_HCD
B_ETD
cross−linker cleavage
A+XL_S
B+XL_L
A+XL_L
B+XL_S
y10
z3
z4
y4
z2
z3
c6
c2
c3
c11
intensity
mass
KIEVVK+XL_S
A_b_ion
A_y_ion
y4+
y1+
y2+
b2+
y3+
prec
b3+
b4+
y5+
b5+
m/z
KIEVVK+XL_L
y3++
b1+
b4+
y5+
b3+
DIFQEIFDKHYK+XL_S
DIFQEIFDKHYK+XL_L
B_b_ion
B_y_ion
y10++
y6+
y7+
b5+
y11++
y5+
y4+
y9+
y8+
b10+
y10+
b11+
b9+
b8+
b7+
prec
